# Dr. William Irving Thornton

**Published:** 1916-02

**Authors:** 


**Dr. William Irving Thornton,** Buffalo 1899, died at his home in Buffalo Jan. 16, aged 37, of pleurisy following pneumonia. He was born in Buffalo Jan. 20, 1879, the son of George H. and Delia Thornton, and the nephew of Dr. William Harvey Thornton. He graduated from the Buffalo Central High School in 1896. He served as interne in the Buffalo General Hospital for 21 months, and then for several months at the German Deaconess Hospital. Since 1901 he had been in practice in Buffalo. He was a member of the organization and local Academy of Medicine, Director of the Lafayette General Hospital and Attendant to the Buffalo Orphan Asylum. He was well and most favorably known both as a practitioner, for his interest in professional activities and as a man.

